# Chloroplast- or Mitochondria-Targeted DEAD-Box RNA Helicases Play Essential Roles in Organellar RNA Metabolism and Abiotic Stress Responses

**DOI:** 10.3389/fpls.2017.00871

**Published:** 2017-05-24

**Authors:** Ghazala Nawaz, Hunseung Kang

**Affiliations:** Department of Plant Biotechnology, College of Agriculture and Life SciencesChonnam National University, Gwangju, South Korea

**Keywords:** chloroplast, DEAD-box RNA helicase, mitochondria, RNA metabolism, stress response

## Abstract

The yields and productivity of crops are greatly diminished by various abiotic stresses, including drought, cold, heat, and high salinity. Chloroplasts and mitochondria are cellular organelles that can sense diverse environmental stimuli and alter gene expression to cope with adverse environmental stresses. Organellar gene expression is mainly regulated at posttranscriptional levels, including RNA processing, intron splicing, RNA editing, RNA turnover, and translational control, during which a variety of nucleus-encoded RNA-binding proteins (RBPs) are targeted to chloroplasts or mitochondria where they play essential roles in organellar RNA metabolism. DEAD-box RNA helicases (RHs) are enzymes that can alter RNA structures and affect RNA metabolism in all living organisms. Although a number of DEAD-box RHs have been found to play important roles in RNA metabolism in the nucleus and cytoplasm, our understanding on the roles of DEAD-box RHs in the regulation of RNA metabolism in chloroplasts and mitochondria is only at the beginning. Considering that organellar RNA metabolism and gene expression are tightly regulated by anterograde signaling from the nucleus, it is imperative to determine the functions of nucleus-encoded organellar RBPs. In this review, we summarize the emerging roles of nucleus-encoded chloroplast- or mitochondria-targeted DEAD-box RHs in organellar RNA metabolism and plant response to diverse abiotic stresses.

## Introduction

The biggest threat to the increasing population worldwide is the scarcity of food due to reducing crop yields caused by various abiotic and biotic stresses such as drought, cold, heat, high salinity, UV, bacteria, fungi, and viruses (Zsigmond et al., 2008; Nouri et al., 2015). As sessile organisms, plants have evolved various strategies to withstand these adverse environmental conditions (Komatsu and Hossain, 2013; Ranty et al., 2016). The survival of plants against these environmental stresses depends on their ability to recognize stress stimuli and adapt to such stresses by regulating the expression of stress-responsive genes in cellular organelles, nucleus, and cytoplasm. Photosynthesis in chloroplasts (Biswal et al., 2011; Ashraf and Harris, 2013; Komatsu and Hossain, 2013) and energy metabolism in mitochondria (Zsigmond et al., 2008) are particularly important cellular processes necessary for plant growth and survival under stressful and normal growth conditions. Therefore, gene expression affecting photosynthesis and energy metabolism in chloroplasts and mitochondria should be tightly regulated for plant growth and survival under stress conditions.

Expression of genes in chloroplasts and mitochondria is regulated mainly at posttranscriptional levels, including RNA processing, intron splicing, RNA editing, RNA turnover, and translational control (del Campo, 2009; Hammani and Giegé, 2014; Sun and Guo, 2016). Although the genomes of chloroplast and mitochondrion harbor less than 150 genes, more than 3,000 and 2,000 nucleus-encoded proteins are transported to the chloroplast and mitochondrion, respectively, and play essential roles in posttranscriptional RNA metabolism in cellular organelles (Millar et al., 2006; Nott et al., 2006; Pesaresi et al., 2007; del Campo, 2009). Therefore, fine-tuning communications between chloroplasts or mitochondria and the nucleus via anterograde signaling and retrograde signaling is essential for organellar gene expression, biogenesis, and function (Zsigmond et al., 2008; del Campo, 2009; Sun and Guo, 2016). Regulation of RNA metabolism in chloroplasts and mitochondria mediated by nucleus-encoded proteins is important for plants to adapt to deleterious biotic and abiotic stresses (Simpson and Filipowicz, 1996; Meierhoff et al., 2003; Williams and Barkan, 2003; Rocak and Linder, 2004; Floris et al., 2009). Many recent studies have demonstrated that diverse RNA-binding proteins (RBPs) play central roles in plant growth, development, and stress responses (Lorković, 2009; Cook et al., 2011; Ambrosone et al., 2012; Kang et al., 2013; Lee and Kang, 2016). Considering that organellar RNA metabolism and gene expression largely depend on nucleus-encoded RBPs, it is imperative to understand the functions of nucleus-encoded organellar RBPs to provide us deeper insights into how plants respond to diverse environmental stresses. Among RBPs are DEAD-box RNA helicases (RHs) that can assist the formation of functional mature RNAs in chloroplasts and mitochondria (Cordin et al., 2006; Kohler et al., 2010). In this review, we will focus on the emerging roles of nucleus-encoded chloroplast- or mitochondria-targeted DEAD-box RHs in organellar RNA metabolism and plant responses to diverse abiotic stresses.

## Structural Features of Dead-Box RNA Helicases

**FIGURE 1:**
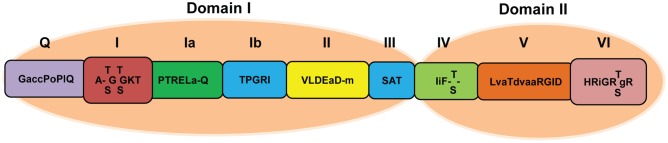
**Schematic representation of the domain structure of SF2 family DEAD-box RH**. The Q, I, Ia, Ib, II, and III motifs are included in domain I, and motifs IV, V, and VI are in domain II. Capital letters represent highly conserved amino acid sequences with >80% homology while small letters indicate less conserved amino acid sequences with 50–80% homology.

RHs are enzymes implicated in a number of cellular processes involving alteration of RNA structures. Based on their shared sequence motifs, RHs are classified into six super families (SF1–SF6). Superfamily II (SF2), the largest helicase family, is mainly composed of DEAD-box RHs (Gorbalenya and Koonin, 1993; Fairman-Williams et al., 2010). Unwinding of double-stranded nucleic acids (DNAs and RNAs) requires energy. Hence, all DEAD-box RHs contain a nucleoside triphosphate (NTP) binding motif. Besides this NTP motif, DEAD-box RHs harbor eight motifs called Q, I, Ia, Ib, II, III, IV, V, and VI (Caruthers and McKay, 2002; Silverman et al., 2003; Tuteja and Tuteja, 2004; Kwong et al., 2005; Singleton et al., 2007) falling under two helicase domains: domain I and domain II. They span over a conserved core region of 400–700 amino acids (de la Cruz et al., 1999; Rocak and Linder, 2004; **Figure 1**). Each motif has specific function for helicase activity. The Q-motif is the most recently discovered motif. It has highly conserved glutamine residue and regulates ATP binding and hydrolysis (Tanner, 2003; Tanner et al., 2003). Motif I (AxxGxGKT) forms a loop structure (P loop) that accommodates ATP and facilitates interactions with Mg^2+^ ions (Tanner and Linder, 2001). Motif Ia forms the groove for facilitating single-stranded DNA/RNA binding (Velankar et al., 1999). Motif II contains a so-called “DEAD” (Asp-Glu-Ala-Asp) box. Its highly conserved first Asp (D) residue interacts with Mg^2+^ ion which is required for NTP binding (Gorbalenya and Koonin, 1989). On the basis of sequence alteration in motif II, SF2 members are classified into three sub-groups: Ski2, DEAD-box, and DEAH-box. Both DEAD-box and DEAH-box RHs have close sequence similarities except alterations in conserved amino acid sequences at motif II (Tanner and Linder, 2001). Motif III (SAT) is required for NTPase and helicase activities and performs the unwinding of RNA (Pause and Sonenberg, 1992). Motif VI (HxxGRxxR) is the third most conserved segment (Gorbalenya and Koonin, 1989). It is part of the ATP-binding cleft and also involved in coupling between helicase and NTPase activities of the protein (Pause and Sonenberg, 1992). The structures of RHs indicate that the remaining motifs (Ib, IV, and V) are probably involved in RNA binding (Rocak and Linder, 2004), although biochemical data are still lacking.

## Occurrence of Dead-Box RHs in the Chloroplasts and Mitochondria of Plants

Although numerous reports have demonstrated that the plant genome encodes a large number of RHs which play significant roles in the regulation of various cellular metabolic pathways, their occurrence and functions in chloroplasts and mitochondria have not been well-documented yet. The genomes of *Caenorhabditis elegans* and *Drosophila melanogaster* encode 30 and 34 DEAD-box RHs, respectively, which are only half of the number of DEAD-box RHs found in plants (Boudet et al., 2001). In *Arabidopsis thaliana*, at least 120 RH members have been predicted using TAIR database^1^ (Mingam et al., 2004; Umate et al., 2010), and 58 DEAD-box RHs have been identified so far (Aubourg et al., 1999; Boudet et al., 2001; Zybailov et al., 2008). Among the identified 58 DEAD-box RHs, 10 DEAD-box RHs target chloroplasts and eight DEAD-box RHs are localized in the mitochondria (**Table 1**). The genome of rice (*Oryza sativa*) harbors more than 60 genes encoding DEAD-box RHs (Umate et al., 2010). Among the identified rice DEAD-box RHs, one RH, OsABP, has been confirmed to be localized in chloroplasts (Macovei et al., 2012), and one RH, OsSUV3, has been confirmed to be localized in mitochondria (Tuteja et al., 2013). To date, functions of only one DEAD-box RH have been determined in the chloroplasts of tobacco (*Nicotiana tabacum*), barely (*Hordeum vulgare*), and maize (*Zea mays*) (**Table 1**).

**Table 1:** List of DEAD-box RHs associated with RNA metabolism in chloroplasts or mitochondria and stress responses in various plants.

Plant	Localization	Gene	Functions	Reference
*A. thaliana*	Chloroplast	RH22	mRNA accumulation during seed growth and development, biogenesis of chloroplast ribosomes, accumulation of TuMV CP	Chi et al., 2012; Kanai et al., 2013
		RH39	Maturation of 23S rRNA in chloroplasts	Nishimura et al., 2010
		AtRH3	Intron splicing, rRNA processing, ABA response	Lee et al., 2013; Asakura et al., 2012; Gu et al., 2014
		AtRH26	Participates in TuMV replication complex, expression modulated by abiotic stresses	Zybailov et al., 2008; Mingam et al., 2004; Umate et al., 2010; Li et al., 2016
		AtRH41	Expression modulated by abiotic stresses	Umate et al., 2010; Li et al., 2016
		ISE2	Group II intron splicing, plasmodesmata regulation	Carlotto et al., 2016
		AtRH47 AtRH50 AtRH52 AtRH58	Expression modulated by abiotic stresses	Zybailov et al., 2008; Mingam et al., 2004; Umate et al., 2010
	Mitochondria	PMHI (AtRH9)	Group II intron splicing Seed germination under salt stress Participates in TuMV replication complex	Matthes et al., 2007 Kim et al., 2008 Kohler et al., 2010 Li et al., 2016
		PMH2	Group II intron splicing	Matthes et al., 2007; Kohler et al., 2010
		AtRH31 AtRH33	Expression modulated by abiotic stresses Participates in TuMV replication complex	Umate et al., 2010; Li et al., 2016
		ABO6	Group I intron splicing, ABA and auxin response	He et al., 2012
		PLT1	Regulation of root meristem activity under ABA	Yang et al., 2014
		MDDX28	Communications between the nucleus and mitochondria	Valgardsdottir et al., 2001
		AtSUV3	ATPases activity, mitochondrial mRNA processing/degradation	Gagliardia et al., 1999
*O. sativa*	Chloroplast	OsABP	Regulation of signal transduction, stress response	Macovei et al., 2012
	Mitochondria	OsSUV3	RNA surveillance and turn over, salinity tolerance	Tuteja et al., 2013; Sahoo et al., 2014
*N. tabacum*	Chloroplast	VDL	Early differentiation of chloroplasts	Wang et al., 2000
*Z. mays*		ZmRH3	Intron splicing, rRNA processing	Asakura et al., 2012
*H. vulgare*		HVD1	Expression modulated by salt stress	Nakamura et al., 2004

In addition to these previously identified DEAD-box RHs, our search for DEAD-box RHs harboring potential target sequences for chloroplasts or mitochondria localization revealed more potential chloroplast- or mitochondria-targeted DEAD-box RHs. Using web-based software and database^2,3,4,5,6^, we identified 12 DEAD-box RHs with putative chloroplast transit peptide (cTP) sequence and four DEAD-box RHs with putative mitochondrial targeting sequences in rice. We also identified seven DEAD-box RHs containing cTP sequences and five DEAD-box RHs harboring mitochondrial targeting sequences in maize. In addition, we found eight DEAD-box RHs with potential cTP sequences and seven DEAD-box RHs having mitochondrial targeting sequences in wheat (*Triticum aestivum*; **Table 2**). These findings suggest that approximately 7–12 DEAD-box RHs and 4–7 DEAD-box RHs are targeted to the chloroplast and mitochondria, respectively, which are much larger numbers than previously reported, indicating that DEAD-box RHs might play significant roles in gene expression regulation and functions in chloroplasts and mitochondria.

**Table 2:** List of predicted chloroplast- or mitochondria-targeted DEAD-box RHs and stress-responsive expression patterns.

Plant	Localization^#^	Gene ID	Modulation^∗^		
			Drought	Cold	Salt
*O. sativa*	Chloroplast	Os01g43120 (RH25), Os01g73900 (RH58) Os01g08930 (RH39), Os03g51900 (OsRH16) Os04g43140 (RH13), Os06g40020(RH52) Os07g05050 (OsRH53), Os08g32090 (RH29)	Down-regulated		
		Os01g07080 (Q761Z9), Os09g34910 (OSRH7)	Up-regulated		
		Os01g07080 (RH18), Os12g05230 (OsRH-like)	No change		
	Mitochondria	Os01g10050 (OsRH20), Os03g19530 (RH24) Os07g45360 (RH57)	Down-regulated		
		Os05g01990 (RH17)	Up-regulated		

			**Drought**	**Cold**	**Heat**
			
*Z. mays*	Chloroplast	GRMZM2G357923 (ZmRH13), GRMZM2G100043 (ZmRH22) GRMZM2G085587 (ZmRH41), GRMZM2G113267 (ZmRH50) GRMZM2G000823 (ZmRH38), GRMZM2G480809 (ZmRH7) AC198169.4_FG004 (ZmRH47)	Down-regulated		
	Mitochondria	GRMZM2G099253 (ZmRH36), GRMZM2G143246 (ZmRH20) GRMZM5G857708 (ZmRH14), GRMZM2G346278 (ZmRH17)	Down-regulated		
		GRMZM2G362850 (ZmRH30)	Up-regulated		

*T. aestivum*	Chloroplast	TRIAE_CS42_5BL_TGACv1_405472_AA1328050 (RH3) TRIAE_CS42_5AL_TGACv1_378036_AA1250890 (RH29)	Down-regulated		
		TRIAE_CS42_5BL_TGACv1_405071_AA1319340 (RH7) TRIAE_CS42_2DL_TGACv1_162281_AA0562360 (RH13) TRIAE_CS42_2AS_TGACv1_112416_AA0337680 (RH36) TRIAE_CS42_4DS_TGACv1_362163_AA1177330 (RH16) TRIAE_CS42_3DL_TGACv1_251106_AA0877450 (RH58) TRIAE_CS42_5AL_TGACv1_377240_AA1245280 (RH28)	No data available		
	Mitochondria	TRIAE_CS42_4AS_TGACv1_307021_AA1016210 (RH24) TRIAE_CS42_3AS_TGACv1_211081_AA0684720 (RH20) TRIAE_CS42_6AL_TGACv1_473376_AA1530620 (RH47) TRIAE_CS42_4DL_TGACv1_344498_AA1147590 (RH17) TRIAE_CS42_4DL_TGACv1_342854_AA1123970 (RH50) TRIAE_CS42_3B_TGACv1_221078_AA0729540 (RH39) TRIAE_CS42_5DL_TGACv1_433272_AA1407810 (RH22)	No data available		

*^#^Ensembl Plants (http://plants.ensembl.org) and NCBI (https://www.ncbi.nlm.nih.gov) were used to search for DEAD-box RHs. Cellular localization of proteins was predicted using TargetP 1.1 Server (http://www.cbs.dtu.dk/services/TargetP), ChloroP 1.1 Server (http://www.cbs.dtu.dk/services/ChloroP), and Predotar Server (https://urgi.versailles.inra.fr/predotar)*.

*^∗^GENEVESTIGATOR (https://genevestigator.com) and BAR (www.bar.utoronto.ca) were used to analyze the stress-responsive expression patterns of each DEAD-box RH*.

## Dead-Box RHs Play Crucial Roles in RNA Metabolism in Chloroplasts and Mitochondria

**FIGURE 2:**
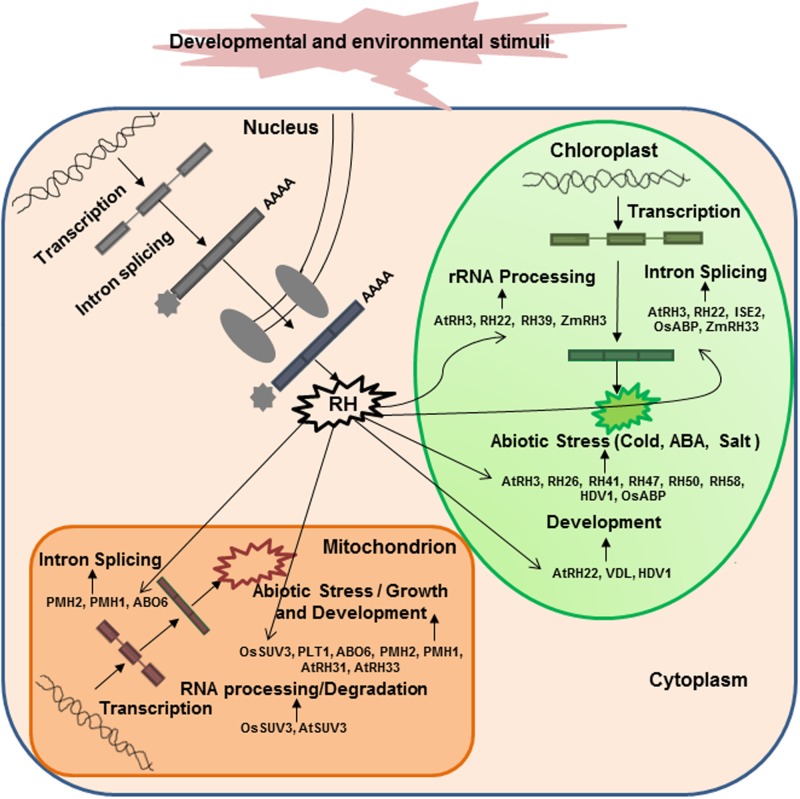
**Cellular functions of diverse DEAD-box RHs involved in RNA metabolism in chloroplasts or mitochondria**. A variety of nucleus-encoded RHs are targeted to chloroplasts or mitochondria and play essential roles in RNA processing, intron splicing, and translation, crucial for organellar biogenesis, function, and stress responses. Examples of the experimentally verified DEAD-box RHs that play unique roles in chloroplasts and mitochondria are shown in parenthesis. The functions and cellular roles of each RH are described in **Table 1**.

Increasing numbers of recent reports have pointed to the importance of DEAD-box RHs in the regulation of RNA metabolism in chloroplasts and mitochondria (**Figure 2**). Arabidopsis RH3, RH22, and ISE2 are involved in the splicing of group I and II introns and the processing of 23S and 16S rRNAs in chloroplasts to help assemble the 50S ribosomal subunit (Chi et al., 2012; Kanai et al., 2013; Gu et al., 2014; Carlotto et al., 2016). In particular, Arabidopsis RH39 has been found to introduce a hidden break in chloroplast 23S rRNA, which is an essential process for the maturation of 23S rRNA (Nishimura et al., 2010). A recent report has demonstrated that chloroplast-targeted ZmRH3 is involved in rRNA biogenesis and the splicing of chloroplast introns in maize (Asakura et al., 2012). Several mitochondria-localized DEAD-box RHs such as ABO6, PHM1, and PHM2 can regulate the splicing of intron-containing genes in Arabidopsis (Matthes et al., 2007; Kohler et al., 2010; He et al., 2012). Mitochondria-localized AtSUV3 and OsSUV3 are involved in mRNA processing and RNA degradation to regulate gene expression in Arabidopsis and rice, respectively (Gagliardia et al., 1999; Tuteja et al., 2013). A mitochondrial MDDX28 is involved in mRNA transport, which is important for communications between the nucleus and mitochondria (Valgardsdottir et al., 2001). Recently, several chloroplast-localized DEAD-box RHs (such as RH58, RH47, RH26, RH50, RH41, and RH52) and mitochondria-targeted DEAD-box RHs (such as RH33 and RH31) have been shown to regulate transcription in response to multiple abiotic stresses (Umate et al., 2010). Moreover, chloroplast-targeted AtRH26 and AtRH41 and mitochondria-localized DEAD-box RHs, AtRH9, AtRH33, and AtRH31 have been found to interact with proteins involved in the formation of replication complex in turnip mosaic virus (Li et al., 2016). All these reports emphasize that DEAD-box RHs are essential for RNA metabolism in chloroplasts and mitochondria.

Although the functions of many DEAD-box RHs in chloroplasts or mitochondria await further experimental verification, their possible cellular roles can be inferred from the studies on nuclear RHs in plants. Two rice DEAD-box RHs, OsRH2 and OsRH34, are essential components of exon junction complex (Huang et al., 2016), and rice TOGR1 (for thermo-tolerant growth required 1) is involved in pre-rRNA homeostasis (Wang et al., 2016). Arabidopsis DEAD-box RHs (AtRH7, AtRH36, and AtRH57) and maize ZmDRH1 play essential roles in rRNA processing (Huang et al., 2010; Hsu et al., 2014; Liu et al., 2016). Notably, the CARPEL FACTORY, an Arabidopsis DEAD-box RH, is involved in the biogenesis of microRNAs (miRNAs; Park et al., 2002) and nuclear DEAD-box RHs play a crucial role in the metabolism of aberrant and silencing RNAs (reviewed in Linder and Owttrim, 2009). Considering that chloroplasts and mitochondria also contain small non-coding RNAs (ncRNAs) and miRNAs (Lung et al., 2006; reviewed in Hotto et al., 2012), it is of keen interest to determine whether DEAD-box RHs are involved in the processing and biogenesis of ncRNAs or miRNAs in chloroplasts and mitochondria.

## Chloroplast- Or Mitochondria-Targeted Dead-Box RHs have Diverse Roles in Plant Growth and Abiotic Stress Responses

Several recent studies have demonstrated that chloroplast- or mitochondria-targeted DEAD-box RHs play essential roles in plant growth and development under normal conditions (**Figure 2**). A chloroplast-targeted RH, INCREASED SIZE EXCLUSION LIMIT 2, is required for group II intron splicing and is involved in chloroplast pigmentation and plasmodesmata regulation during embryogenesis of Arabidopsis (Carlotto et al., 2016). A tobacco DEAD-box RH, VDL (for variegated and distorted leaf), is involved in early chloroplast maturation and regulates flower and root growth (Wang et al., 2000). Several mitochondria-localized RHs, PMHI (AtRH9), PMHII, and ABO6, regulate seed germination in Arabidopsis (Kim et al., 2008; Kohler et al., 2010; He et al., 2012). These results clearly show that chloroplast- or mitochondria-targeted DEAD-box RHs play important roles in plant growth and development. As nuclear DEAD-box RHs are involved in regulating programmed cell death in rice (Li et al., 2011) and two DEAD-box RHs in rice, OsRH2 and OsRH34, are necessary for pollen and seed development (Huang et al., 2016), further determination of the functions of chloroplast or mitochondrial DEAD-box RHs in plant development is necessary.

Although the functions of chloroplast- or mitochondria-targeted DEAD-box RHs in plant growth and development are known for only a few cases, potential roles of DEAD-box RHs in abiotic stresses are increasingly being discovered (**Table 1** and **Figure 2**). Chloroplast-localized RH3 (Gu et al., 2014) and mitochondria-targeted PMHI (AtRH9; Matthes et al., 2007; Kim et al., 2008; Kohler et al., 2010) have been found to confer freezing tolerance in Arabidopsis. A chloroplast-localized HVD1 has been found to be up-regulated by cold and salt stress and affected photosynthetic activity under stress in barley (Nakamura et al., 2004). A chloroplast-localized OsABP is involved in the response of rice to diverse abiotic stresses (Macovei et al., 2012). Several studies have demonstrated that mitochondria-localized rice OsSUV3 and Arabidopsis AtOsSUV3 can regulate the expression of various stress-induced genes and confer plants tolerance to salt stress (Gagliardia et al., 1999; Tuteja et al., 2013; Sahoo et al., 2014). Recently, mitochondrial PLT1 (PLETHORA1) and ABO6 (ABA overly sensitive) have been found to play important roles in the regulation of primary root growth and root meristem activity by modulating ABA and auxin signaling (He et al., 2012; Yang et al., 2014). A chloroplast-targeted RH3 is reported to be essential for carbon fixation and the maintenance of ABA level in Arabidopsis under environmental stresses (Lee et al., 2013).

In addition to these already-verified chloroplast- or mitochondria-targeted DEAD-box RHs, our analysis using GENEVESTIGATOR^7^ and Bio-Analytic Resource for Plant Biology^8^ servers showed that the expression of potential chloroplast- or mitochondria-targeted DEAD-box RHs found in rice, maize, and wheat was high modulated by various abiotic stresses (**Table 2**), implying that more DEAD-box RHs in chloroplasts or mitochondria might be involved in abiotic stress responses. Interestingly, the expression of majority of chloroplast- or mitochondria-targeted DEAD box-RHs in rice, maize, and wheat is down-regulated under diverse abiotic stresses (**Table 2**). Although the physiological significance of such stress-responsive expression patterns of DEAD-box RHs remains unclear, these findings suggest that a large number of nucleus-encoded chloroplast- or mitochondria-targeted DEAD-box RHs might be involved in plant responses to diverse abiotic stresses. Considering that photosynthesis in chloroplasts functions as a global sensor of abiotic stresses (Biswal et al., 2011) and that expression of genes in chloroplasts is mainly regulated at posttranscriptional RNA metabolism (del Campo, 2009; Stern et al., 2010), it is interesting to determine how DEAD-box RHs affect the processing, splicing, and decay of chloroplast transcripts involved in photosynthesis, which will provide further insights into the importance of DEAD-box RHs in plant growth and survival under stress conditions.

## Concluding Remarks and Future Perspectives

Although recent progress on the analysis of plant genomes and proteomes has revealed the presence of a large number of DEAD-box RHs targeted to chloroplasts or mitochondria, cellular roles of DEAD-box RHs in organellar RNA metabolism and function remain unclear. It is evident that DEAD-box RHs targeted to either chloroplasts or mitochondria is crucial for the regulation of gene expression and RNA metabolism in these cellular organelles. However, more analyses are needed to determine the functions of each DEAD-box RH family member. Considering that communications between the nucleus and chloroplasts or mitochondria via anterograde and retrograde signaling are essential for organellar gene expression, biogenesis, and function (Zsigmond et al., 2008; del Campo, 2009; Sun and Guo, 2016), it would be of great interest to determine whether chloroplast- or mitochondria-targeted DEAD-box RHs can affect the expression of nuclear genes under stress conditions. In addition, it is necessary to determine the mechanistic roles of DEAD-box RHs in organellar RNA metabolism. Given that several RHs function as RNA chaperones to aid RNA folding via structural rearrangement of substrate RNAs (Kang et al., 2013; Lee and Kang, 2016), it is likely that these chloroplast- or mitochondria-targeted DEAD-box RHs might also function as RNA chaperones in organellar RNA metabolism. Major future tasks should be focused on identifying RNA targets and understanding how DEAD-box RHs recognize substrate RNAs to regulate posttranscriptional RNA metabolism in cellular organelles. Such studies will provide further insights into the importance of DEAD-box RHs targeted to chloroplasts and mitochondria in plant growth and survival.

## Author Contributions

GN and HK contributed equally writing the review.

## Conflict of Interest Statement

The authors declare that the research was conducted in the absence of any commercial or financial relationships that could be construed as a potential conflict of interest.

## Footnotes

1 http://www.arabidopsis.org

2 http://plants.ensembl.org

3 http://www.ncbi.nlm.nih.gov

4 http://archive.plants.ensembl.org/Triticum_aestivum/Info/Index

5 http://ensembl.gramene.org/Triticum_aestivum/Info/Index

6 http://ensembl.gramene.org/Zea_mays/Info/Index

7 https://genevestigator.com

8 http://www.bar.utoronto.ca
